# Safety and feasibility of En‐bloc holmium laser enucleation for very large prostates (> 200 cc) with trainee involvement

**DOI:** 10.1002/bco2.469

**Published:** 2024-12-20

**Authors:** Feres C. Maluf, Ansh Bhatia, Archan Khandekar, Diana M. Lopategui, Joao G. Porto, Ryan R. Chen, Jean C. Daher, Mohamadhusni Zarli, Robert Marcovich, Hemendra N. Shah

**Affiliations:** ^1^ University of California San Francisco San Francisco CA USA; ^2^ Miller School of Medicine Desai Sethi Urology Institute, University of Miami Miami FL USA; ^3^ Dr. Kiran C. Patel College of Osteopathic Medicine Nova Southeastern University Fort Lauderdale FL USA

**Keywords:** benign prostatic hyperplasia (BPH), En‐bloc, holmium laser enucleation

## Abstract

**Objectives:**

To evaluate the safety and feasibility of “en‐bloc” Holmium Laser Enucleation of the Prostate (HoLEP) with trainee involvement in patients with prostates larger than 200 cc.

**Patients and Methods:**

A retrospective analysis was conducted on patients undergoing HoLEP using the “en‐bloc” technique for prostate sizes > 200 cc between July‐2017 and December‐2023 at an academic teaching hospital. Perioperative data was collected, including patient demographics, clinical parameters, operative details and functional outcomes. Patients who continued to experience incontinence at 1 year were further followed up at 2 years to update their continence status. Sub‐group analysis was performed to compare outcomes between patients with preoperative prostate size of 200–300 cc and > 300 cc.

**Results:**

The analysis included 89 patients with a mean age of 73.12 ± 8.10 years. Preoperative prostate weight ranged from 200 to 401 cc with a median of 245 cc, and median PSA was 7.71 ng/ml. Median operative time was 218.5 minutes, and median enucleated prostate volume was 164.2 cc. Median postoperative PSA was 0.4 [0.21–0.78] ng/ml. At 1‐year follow‐up, mean IPSS was 1 ± 2.4, Qmax was 27.03 ± 11.57 ml/s and PVR was 21.6 ± 28.6 ml. Postoperative complications included blood transfusion (5.6%), acute renal injury (4.5%), urinary tract infection (2.2%), postoperative urinary retention (2.2%) and urethral stricture (5%). Although transient urinary incontinence was noted in 41.6% at 1–3‐months, complete continence was achieved in 83.3% and 96.3% at 1 and 2 years postoperatively, respectively. Subgroup analysis showed significant differences in operative time and enucleated weight between prostates 200–300 cc and > 300 cc, but no significant differences in postoperative IPSS, PVR or Qmax at 3‐months.

**Conclusion:**

“En‐bloc” HoLEP is a feasible and safe procedure for prostates larger than 200 cc, demonstrating favourable perioperative and functional outcomes despite the extended operative times and involvement of trainees.

## 1 INTRODUCTION

For prostate glands > 200 cc, preferred surgical options are typically simple prostatectomy and holmium laser enucleation of the prostate (HoLEP) due to their efficacy in managing large volumes of prostate tissue. Due to its minimally invasive nature, HoLEP is the preferred modality for managing these large prostates (1). The first HoLEP technique described was the three‐lobe approach, which entails making three incisions at the bladder neck that are carried deep to the level of the prostatic capsule fibres and then distally to the level of the verumontanum.^1^, ^2^, ^3^ An alternative developed was the two‐lobe approach, which dissects the lateral lobe on one side and the lateral with the medial lobe on the other, reducing one bladder neck incision. More recently, the “En Bloc” technique was described, which is performed by enucleating all the lobes together, starting from the verumontanum and progressing proximally towards the bladder neck.^2^, ^3^

Several studies have compared these techniques without clear evidence favouring one over the others. In a recent retrospective comparative trial, the outcomes were similar except for a faster enucleation time in the modified two‐lobe method and higher rates of transient urine leakage in the “en bloc” approach. However, other studies showed that the “en bloc” had the fastest time‐efficient enucleation, reaching a difference as high as 30%.^3^, ^4^, ^5^

Despite HoLEP being a size‐independent procedure, prostates > 150 cc possess unique technical challenges. For instance, maintaining the plane of dissection might become more challenging due to difficulty in localizing anatomic landmarks, especially in the presence of “satellite” adenomas. Higher vascularity of large prostates could potentially increase the risk of intraoperative bleeding, thereby affecting endoscopic visualization.^6^, ^7^ Additionally, urologists who have adopted the “en‐bloc” technique still prefer the 2‐ or 3‐lobe technique for large glands due to anticipated difficulty pushing the large, enucleated adenoma into the bladder for morcellation. Although there are reports of the safety and feasibility of HoLEP in prostates > 200 cc, most authors employed two‐ or three‐lobe techniques (Table 1).^6^, ^8^, ^9^, ^10^, ^11^, ^12^, ^13^, ^14^, ^15^

**TABLE 1:** Literature review of similar studies assessing the HoLEP procedure in very large prostates.

Study, year	Technique	Population (N)	Age (years)§	Preoperative PSA (ng/mL)§	Postoperative PSA (ng/mL)§	Baseline PV (g)§	Operative time (min)§	Enucleated PV (g)§	Transient TUI†	Blood transfusions†	Follow‐up (months)
Porto et al, 2023^8^	En bloc	43 (> 200 g)	72.3 (± 9.1)	7.7 (4.8–12.4)	0.4 (0.2–0.5)°	232 (210–270)	199 (150–263)	155 (117–203)	10 (23.3)°	3 (7)	12
Tricard et al, 2023^11^	3‐lobe	81 (≥ 150 g)	73.9 (±7.3)	14.8 (±11.6)	0.8 (±0.5)*	183.3 (±34.5)	57.5 (±29.7)	151.8 (±44.7)	2 (2.5)*	2 (2.5)	6
Vand der Jeugt et al, 2023^12^	3‐lobe	22 (> 200 g)	77.5 (72–82)	7.5 (7.0–11.0)	0.4 (0.3–0.4)^¡^	204.5 (204–224)	132.5 (120–155)	180 (151–202)	4 (18)°	0	12
Tay et al, 2022^13^	3‐lobe*	97 (≥ 150 g)	71.5 (±8.3)	13.2 (±11.7)	3.9 (±12.2)°	195.3 (±49.1)	86 (±35)ª	124 (±46)	23 (23.7)°	2 (2)	3
Assmus et al, 2021^14^, ^œ^	3‐lobe/ 2‐lobe	55 (≥ 175 g)	73.8 (56–91)	8.58 (2.70–15.66)	0.87 (0.08–3.25)°	229.9 (175–535)	121.6 (37–243)	165.1 (45–385)	‐	‐	3
Boxall et al, 2021^9^	3‐lobe*	157 (> 200 g)	73 (67–77)	‐	‐	4/3 of enucleated volume	70 [60–90]^¶^	190 (168–223)	17 (10.8)°	7 (4.5)	3
Zell et al, 2021^10^	3‐lobe/ 2‐lobe	88 (≥ 200 g)	73.2 (±7.7)	15.5 (±12.1)	1.47 (±2.46)*	253 (±74)	172.2 (±55.7)	159.7 (±79.9)	‐	17 (19.3)	3
Kim et al, 2015^6^	4‐lobe/ 3‐lobe	6 (> 200 g)	72.7 (±9.9)	12.84 (±3.22)	‐	252.1 (±59.5)	76.7 (±19.6)^¶^	117.9 (±84.0)	2 (33.3)*	0	6
Krambeck et al, 2010^15^, ^œ^	3‐lobe/ 2‐lobe	57 (> 175 g)	72 (48–90)	14.6 (1.6–48)	0.78 (0.01–2.5)*	217.8 (174.6–391)	91.9 [30–263]^¶^	176.4 (48–532.2)	‐	2 (3.5)	6

HoLEP: Holmium Laser Enucleation of the Prostate; PSA: Prostate‐specific antigen; PV: Prostate volume; TUI: Transient Urinary Incontinence;

^œ^ Reported as mean (range);

^§^ SD (±); IQR (−);

¶ Enucleation time;

ª Laser time; (%);

° at 3 months;

* at 6 months; time frame not reported.

A previous study by our group demonstrated the feasibility and safety of the “En Bloc” technique in prostates of varying sizes, including those > 200 cc.^8^ In this present study, we aim to focus on the safety and efficacy of HoLEP in patients with prostates > 200 cc utilizing the “En Bloc” technique in the setting of an academic teaching hospital with the involvement of trainees.

## 2 METHODOLOGY

With Institutional Review Board approval (20180511), a retrospective analysis of patients from a prospectively maintained institutional database of HoLEP patients treated between June 2017 and December 2023 was performed. Inclusion criteria were patients undergoing HoLEP with prostate sizes > 200 cc, as determined by preoperative transrectal or transabdominal ultrasound, computerized tomography scan or magnetic resonance imaging (MRI). Patients with prior prostate or pelvic radiation or a known diagnosis of prostate cancer were excluded.

Preoperative evaluation included International Prostate Symptom Score (IPSS), maximum urinary flow rate (Qmax) and postvoid residual urine volume (PVR) for patients who were not in urinary retention. Each patient had a urine culture and prostate‐specific antigen (PSA) measurement before surgery. Patients with positive urine cultures were treated with appropriate antibiotics before surgery. All others received prophylactic antibiotics preoperatively and were continued for 5–7 days postoperatively. Patients with elevated PSA measurement underwent further evaluation with a 4Kscore and/or multiparametric MRI preoperatively. A prostate biopsy was performed after shared decision‐making. Patients on anticoagulants or antiplatelet therapy were managed individually in consultation with their primary care provider, cardiologist or neurologist for adequate cessation of medications preoperatively.

All procedures were performed using the “En‐bloc” technique under general anaesthesia by a single experienced surgeon with the active participation of urology residents and endourology fellows. At our institution, trainees are granted graduated autonomy for all procedures. HoLEP training at our institution involved stepwise graduated progression of the trainee based on proficiency to perform a certain surgical technique. Case complexity matched the experience of the trainee. To elaborate, residents and fellows observed a few cases performed by the expert and then were given simple steps like dissecting the lateral lobe from the capsule. Multiple residents were involved, and once trained in some steps, residents or fellows would also act as trainers for novice trainees. They advanced to more complex steps like performing an initial incision in front of the verumontanum to enter the plane of dissection, dissecting the prostate from the bladder neck, performing posterior dissection, and lastly, apical mucosal release. They would advance in the steps based on their proficiency under the guidance of the expert. Once they had completed all the steps for different patients, they were allowed to complete the case independently with the presence of an expert observing the procedure in the operating room.

The “En‐bloc” technique involves enucleating all prostate lobes together, starting from the verumontanum and progressing proximally towards the bladder neck.^16^ The procedure was performed using a high‐power (100 W) holmium laser machine or its Moses pulse modulation (MOSES™ 1.0 or MOSES™ 2.0) and a 550‐μm laser fibre (Boston Scientific, Marlborough, MA, USA). Laser settings for enucleation were standardized across all cases at 2 J and 30 Hz. Morcellation was performed with a Versacut or PIRANHA morcellator (Richard Wolf, Chicago, IL, USA). Postoperative management involved overnight observation with continuous bladder irrigation to prevent clot retention, and catheters were typically removed the following morning before discharge from the hospital. Anticoagulant/antiplatelet medications were restarted after the procedure.

Data collected included patient demographics (age, body mass index, comorbidities), preoperative clinical parameters (PSA, prostate volume), intraoperative details and postoperative outcomes (catheterization duration, length of hospital stay, complications). Operative time was calculated as the total time spent by the patient in the operating room. The enucleated prostate volume was calculated by the pathologist on receipt of tissue for histopathological examination. Postoperative complications were described according to the Clavien‐Dindo Classification (CDC). Functional outcomes were assessed using the IPSS, Qmax and PVR at 3 months, 6 months and 1 year postoperatively. Postoperative PSA was done approximately 3 months after surgery. Incontinence was defined as urinary leakage of any severity needing protection or pads at any time postoperatively. Descriptive statistics were calculated, with continuous variables presented as medians with interquartile ranges, while categorical variables were summarized as frequencies and percentages. Pearson's correlation coefficient was used to assess the relationship between prostate size and operative time. A subgroup analysis was performed comparing the outcome between patients with preoperative prostate size of 200–300 cc and those > 300 cc. Comparative analyses were performed using the student's t‐test or Wilcoxon signed‐rank test for continuous variables as appropriate and the Chi‐square test for categorical variables. A p‐value < 0.05 was considered statistically significant. All statistical analyses were performed using RStudio software version 4.3.1 (RStudio Inc, Boston, MA).

## 3 RESULTS

The analysis included 89 patients with a mean age of 73 years (Table 2). The preoperative prostate volume ranged from 200 to 401 cc with a median of 245 [217–290] cc, and the median PSA was 7.71 ng/ml. Use of anticoagulants, catheter dependence before surgery and associated bladder calculi was observed in 23.6%, 48.31% and 3.5% of cases, respectively. The median operative time was 218.5 [153.5–271.75] minutes, with postoperative catheter duration and hospital length of stay averaging one day. The median postoperative PSA was 0.4 [0.21–0.78] ng/ml, and the median enucleated prostate volume was 164.2 [131–210] g. Two patients were incidentally found to have prostate cancer during histopathological examination who had Gleason grade 3+4 and 4+3, respectively. A positive correlation (p < 0.002) was observed between prostate size and operative surgical time (Table 2).

**TABLE 2:** Baseline and demographic characteristics and perioperative outcomes.

	Total (n = 89)	200–300 cc group (n = 68)	> 300 cc group (n = 21)	p‐value
**Age (years)**	73.12 ± 8.10	73.6 ± 7.93	71.48 ± 8.64	0.31
**Preoperative PSA**	7.71 [5.03–11.8]	7.31 [4.71–10.6]	10.79 [9.24–13.76]	0.018
**Prostate Volume (g)**	245 [217–290]	223.5 [210.75–253.5]	330 [307–360]	< 0.001
**Use of Anticoagulants/antiplatelet**	23.6%	19 (27.95%)	2 (9.5%)	0.14
**Catheter Dependence**	43 (48.31%)	30 (44.11%)	13 (61.90%)	0.212
**Median Operative Time (mins)**	218.5 [153.5–271.75]	199.5 [150–258]	273.5 [232.5–311.5]	0.002
**Median Enucleated Weight (g)**	164.2 [131–210]	155.4 [128.5–201.1]	204 [149.4–256]	0.022
**Median Catheterization (days)**	1 [1–1]	1 [1–1]	1 [1–1]	0.503
**Median Hospital Stay (days)**	1 [1–1]	1 [1–1]	1 [1–2]	0.721
**Median Postoperative PSA**	0.4 [0.21–0.78]	0.345 [0.15–0.50]	0.46 [0.22–0.75]	0.260

PSA: Prostate‐specific antigen. Values are Median (IQR).

In a subgroup analysis, the median operative time was 199.5 minutes [150–258] for the 200–300 cc group and 273.5 minutes [232.5–311.5] for the > 300 cc group (p = 0.002). The median enucleated prostate volume was 155.4 cc [128.5–201.1] for the 200–300 cc group and 204 cc [149.4–256] for the > 300 cc group (p = 0.022). The median postoperative PSA was 0.345 ng/ml [0.15–0.50] in the 200–300 cc group and 0.46 ng/ml [0.22–0.75] in the > 300 cc group (p = 0.26).

Functional outcomes at 3 months postoperatively improved significantly from baseline (Table 3A). The median IPSS decreased from 18 [11.25–22] to 2 [0.25–4.75] (p < 0.001). The median PVR decreased from 109.5 [45.5–237] ml to 14 [3.5–47] ml (p < 0.001). At the 3‐month mark, subgroup analysis showed no significant difference in IPSS between the 200–300 cc and the > 300 cc groups (p = 0.54). Similarly, there were no significant differences in PVR or Qmax between the subsets at 3 months (Table 3B).

**TABLE 3A:** Voiding outcomes of all included patients.

Variable	Baseline	3‐months	6‐month	1‐year
**IPSS**	18 [11.25–22]	2 [0.25–4.75]	1.5 [0–3]	0 [0–2]
**p‐value (IPSS at timepoints vs baseline)**		< 0.001	< 0.001	< 0.001
**PVR**	109.5 [45.5–237]	14 [3.5–47]	7 [0–32]	11 [0–30]
**p‐value (PVR at timepoints vs baseline)**		< 0.001	< 0.001	< 0.001
**Qmax**	6.4 [5–9.1]	20.05 [11.62–33.75]	22 [13.97–33.10]	25.1 [16.7–36.7]
**p‐value (Qmax at timepoints vs baseline)**		< 0.001	< 0.001	< 0.001

IPSS: International Prostate Score System; Qmax: Peak urinary flow; PVR: Postvoid urinary volume.

**TABLE 3B:** Subgroup comparison at 3‐months.

Variable	200–300 cc group	> 300 cc group	p‐value
**IPSS – Baseline**	18.5 [14–24.25]	14 [9.25–20.25]	0.052
**IPSS – 3mo**	2 [0–4]	2 [1–6]	0.54*
**PVR – Baseline**	105 [35–255]	189 [82–234]	**0.49** *
**PVR – 3mo**	19 [5–51]	5 [0–7]	0.14
**Qmax ‐ Baseline**	6.4 [5–9.5]	5.7 [4.2–6.9]	0.50
**Qmax – 3mo**	19.6 [11.6–31.5]	28.85 [19.5–37.7]	0.42

* Between‐group comparison at 3 months.

Four patients developed intra‐operative complications: shock in the recovery period (needing inotropes for 1 night), aspiration pneumonia (needing intubation overnight), transient fluid overload and bladder mucosal injury with no sequelae. Postoperative complications were also analysed, with 43.8% of the sample presenting with CDC grade I complications, composed of transient urinary incontinence (TUI) and urinary retention. Complications of higher grade were blood transfusion requirement, urinary tract infection, urethral stricture and acute renal injury, with an event rate of 5.6%, 2.2%, 5% and 4.5%, respectively (Table 4). There was no significant difference in the rates of these complications between the subgroups. TUI occurred in 41.6% of patients at a 1–3‐month follow‐up period. Among 54 patients with over 1 year of follow‐up data, complete continence was achieved in 83.3% and 96.3% at 1 and 2 years postoperatively, respectively. Two patients (3.7%) did not achieve continence and remained dependent on 1–2 pads/day for stress urinary incontinence. The median time to achieve continence was 2.64 months, with an interquartile range from 1.6 to 7.4 months.

**TABLE 4:** Postoperative complications.

Clavien‐ Dindo classification	Frequency (%)
Grade I	
Transient Urinary Incontinence	41.6
Grade II	
Blood Transfusion Requirement	5.6
Urinary Tract Infection	2.2
Grade IIIA	
Urethral Stricture	4.5
Urinary Retention	2.2
Grade IVa	
Acute Renal Injury	4.5

## 4 DISCUSSION

The absolute burden of BPH has risen substantially over the last decades, with a 70.5% increase in prevalence from 2000 to 2019.^17^ Many patients manage LUTS secondary to BPH with medical treatment that does not affect the natural progression of the disease for extended periods.^10^ As prostatic volume increases by 2–2.5% annually, this can contribute to the incidence of patients seeking surgery when they develop bothersome LUTS and complications with large underlying prostates.^18^, ^19^ Hence, assessing safe and effective treatments for prostates > 200 cc is paramount. In this study, our group was among the first to share the experience of the “En Bloc” technique with trainee involvement for HoLEP surgery on prostates > 200 cc. The evidence suggests that the procedure is safe and feasible, demonstrating excellent perioperative and long‐term functional outcomes in our patient cohort, even though longer operative times were observed as prostate size increased.

While the “En Bloc” technique is praised for its shorter procedure time than the two‐ and three‐lobe techniques, its efficiency might not hold for cases with bulkier glands.^20^, ^21^, ^22^ We reported one of the most prolonged surgery durations compared to similar studies, and one contributing factor may be the involvement of trainees. Considering other trials that reported trainee involvement, Zell et al. retrospectively reviewed the three‐lobe or two‐lobe techniques on 88 patients with > 200 cc prostates. Their reported mean duration was 172.2 minutes, approximating our reported median of 207 minutes.^10^ Additionally, we established the operation time as the total time spent by the patient in the operating room, while other authors have not reported their definition of surgical duration. They might have reported only surgical duration rather than including the peri‐procedure anaesthesia time. Further comparative trials would be necessary to estimate the difference in procedure time based on a clear description of surgical length calculation, technique and trainee involvement.

We also found a positive correlation between prostate size and operative time. This result aligns with our previous study assessing the “En Bloc” technique in three groups of BPH patients of different sizes. There was a significant stepwise increment in the procedure duration in the groups with larger glands, going from a median of 105 minutes in the group with BPH ≤ 40 cc to 199 minutes in the group with prostates > 200 cc.^8^ Despite this study's longer operative time, the “En Bloc” technique yielded favourable perioperative outcomes.

Specific risks associated with the management of larger prostates include an increased need for cystotomy or conversion to open prostatectomy. Reasons for this include difficulty in completing enucleation or morcellation, whether due to hard nodules, insufficient length of the instruments or the lack of space in the bladder to proceed safely. This may require methods such as a cystotomy or a perineal urethrostomy to remove the enucleated tissue.^6^, ^7^, ^8^, ^9^ Regarding conversion to open prostatectomy, a retrospective analysis by Shvero et al. found prostate size was the only predictive risk factor. When stratified according to a requirement for open conversion, the median prostate volume was 228 cc for the patients who required it versus 95 cc for those who did not.^23^ The need for cystotomy or open prostatectomy conversion was null in our series, while previous cohorts had a 5.7–8% rate of cystotomy for prostates ≥ 200 cc. Moreover, three patients from Zell et al. had to convert to open surgery due to equipment failure, poor visualization of the ureteral orifices and a 770 cc prostate.^9^, ^10^ This constitutes another argument favouring the feasibility and safety of the “En Bloc” technique in this patient population.

BPH procedures are commonly performed in the inpatient setting, with planned overnight observation. Same‐day discharge and same‐day catheter removal have notoriously been successful after HoLEP in many groups, although it is usually reserved for selected patients.^24^ Assmus et al. attempted same‐day discharge in patients with glands ≥ 175 cc. Of 55 patients undergoing HoLEP, 45 were scheduled for same‐day discharge, accomplished in 38 (85%) of them. The hospital stay was 11.8 hours, with an average catheterization time of 21.2 hours.^14^ Considering our population with prostates ≥ 200 cc, a median length of 1 day for both catheter duration and hospital stay seems acceptable. In comparison, Van der Jeugt et al. retrospectively compared the three‐lobe HoLEP technique with robotic‐assisted simple prostatectomy in glands ≥ 200 cc. In the HoLEP group of 22 patients, median catheterization time and length of hospital stay were 2 and 3 days, respectively.^12^

Several studies investigated PSA as a surrogate to measure the efficacy of BPH surgery. A review by Bhat et al. noted that the PSA nadir at 3 to 6 months after the procedure could have an inverse relationship with the magnitude of postoperative improvement in voiding function and its durability.^25^ Our cohort's median postoperative PSA of 0.4 ng/ml is among the lowest in the literature. Compared to studies with similar preoperative PSA, such as Van der Jeugt et al., post‐operative median PSA was significantly higher at 0.8 ng/ml.^10^, ^11^, ^12^, ^13^ Lower PSA nadir in the en bloc technique, as noted in our study compared to other studies, may indicate complete adenoma removal. However, due to a lack of direct comparison, the role of other factors responsible for PSA differences needs to be further investigated.^21^ These findings demonstrate the efficiency of the “En Bloc” technique in extremely large prostates.

Another important outcome is the incidental diagnosis of prostate cancer (iPCa), which occurred in 3.5% of our cohort. A retrospective review of 777 BPH patients undergoing HoLEP yielded an iPCa prevalence of 7.1%, of which 61.8% was Gleason Grade Group 1 (GG 1). In this analysis, larger prostates were a protective factor, representing a 13% reduction in malignancy risk for every 10‐cc increase in prostate size, which aligns with our reportedly lower rates. Conversely, older age and smaller glands were associated with higher‐grade (≥ GG 2) pathology.^26^ Another study by Kizilkan et al. analysing 430 BPH patients undergoing open prostatectomy noted a prevalence of cancer of 5.6%, of which 87.5% was GG 1.^27^ Given the low percentage of patients with iPCa after BPH surgery and most of them having low‐grade disease, we advocate for standard cancer screening recommendations in patients undergoing BPH surgery. However, a postoperative PSA between 3 and 6 months is part of our standard follow‐up to assess surgery efficacy and cancer risk.

Regarding functional outcomes, our cohort demonstrated excellent improvement in all parameters. At 1‐year follow‐up, the mean IPSS was 1, comparable with the median IPSS of 3 in Van der Jeugt's trial. In our group, the median PVR at 1 year was 11, while Van der Jeugt's study had a median of 0 ml.^12^ Once again, to establish differences in outcomes between HoLEP techniques for prostates > 200 cc, randomized controlled trials would provide the best evidence. Additionally, it is important to underscore the heterogeneity of follow‐up periods and functional outcomes between trials, which hinders the literature comparison. Of nine trials with similar populations and interventions, only three had IPSS, Qmax and PVR assessed at 12 months.^6^, ^10^, ^11^, ^12^, ^13^, ^14^, ^15^

The main complications reported in our study were TUI, followed by blood transfusion. Other significant complications were limited to 1 or 2 patients, which included acute renal injury, post‐operative urinary retention, urinary tract infection and urethral stricture. These low rates are comparable to those reported in trials utilizing the three‐lobe technique in extremely large prostates.^6^, ^13^, ^15^

Regarding blood transfusion, a large multi‐centre study by Romero‐Otero et al. examined predictive factors for blood loss in 963 HoLEP patients with a median prostate volume of 102 c. The percentage of participants using anticoagulant/antiplatelet medications was 39%, and the peri‐operative blood transfusion rate was 5%. They found a significant correlation between anticoagulant/antiplatelet use and blood transfusion rates. In addition, operative time was associated with decreased haemoglobin levels, although not with transfusion rates.^28^ Our cohort presented a 26.3% use of anticoagulant/antiplatelet and a 5.6% transfusion rate. Despite a slightly higher transfusion rate with a lower prevalence of anticoagulation use, larger prostates and longer operative time may account for this difference, as these are known risk factors for increased bleeding.^28^

Additionally, one patient had pre‐operative anaemia, which would reduce the blood transfusion rate to 5.2% if not included. As previously stated by our group, patients with severely enlarged prostates should be counselled regarding the increased risk of blood transfusion.^8^ However, the heterogeneity of protocols for blood transfusions may affect the interpretation of these findings, especially in single‐institution studies such as ours. Multi‐institutional studies analysing objective parameters for blood loss, such as haematocrit and haemoglobin decrease, are needed to overcome this limitation.

TUI is a well‐established, bothersome adverse event after HoLEP. Several groups have analysed its possible risk factors. We noted a high rate of TUI (41.6%) during 1–3‐month follow‐up period. Like our series, Elsaqa et al. analysed 666 patients with a median prostate weight of 89 cc, reporting TUI in 43% of them. The features that increased the risk of TUI up to three months post‐procedure were obesity, frailty and larger prostate volume.^29^ Other studies reported a lower rate of TUI (Table 1). This difference is likely due to a lack of a uniform method of reporting post‐operative incontinence. We adhere to rigid criteria of lack of incontinence or use of pads to report continence. Additional trials would be required to compare TUI rates in patients undergoing “En Bloc” HoLEP with other approaches in this specific scenario.

As previously mentioned, the retrospective single‐centre design and the lack of a comparison control group constitute some of the limitations of our study. One example is the lack of standardized clinical workflow, such as the different imaging types used for preoperative volume calculation or the equipment choice for laser enucleation. Prostates over 200ccs needing surgical management are uncommon, explaining our case load for such patients. Other studies, such as Krambeck et al., have 59 patients over 9 years, and Boxall et al. did 157 cases per 15 years (Table 1). We believe that the active involvement of trainees is necessary in such challenging cases so that when they become independent practitioners, they will be confident in their ability to operate in such cases.

Our study reports total operative time instead of procedure time; confounding factors such as anaesthesia issues can affect the total operating time. In addition, we could not assess enucleation and morcellation y. Boxall et al. reported a positive correlation between prostate volume and enucleation efficiency, although it is insignificant for morcellation efficiency.^9^ Enikeev et al. found higher morcellation efficiency with the two‐lobe technique than the “En Bloc” approach in prostates > 150 cc, demonstrating how these variables could provide further insight into our results. Despite that, our study has unique features that should be highlighted.^4^ To our knowledge, this paper is the first in the literature focusing solely on the “En Bloc” technique for prostates above 200 cc, demonstrating its feasibility and safety for this subset of patients. Moreover, the participation of trainees in this specific subset of patients did not affect the feasibility and safety of the procedure, with results comparable to the literature. Therefore, we believe that involving trainees in these complex surgeries in academic institutions is paramount, and that training future HoLEP practitioners can be accomplished without compromising procedure outcomes. Nevertheless, caution is warranted for patients who cannot withstand lengthy procedures, although peri‐operative outcomes and intra‐operative complication rates were advantageous with the “En Bloc” technique.

Although the trainees were involved in all surgeries, the limitation of our study is that it lacks granular data on specific steps in each surgery that was performed by them. HoLEP training at our institution involved stepwise graduated progression of the trainee based on proficiency to perform a certain surgical technique. Case complexity matched the experience of the trainee. Such proficiency‐based progression training has been previously described as safe in advanced robotic surgeries.^30^ We agree with other authors that such a curriculum provides increasing opportunities for urology residents and fellows with an aptitude for HoLEP and can equip them with skills and confidence before entering real‐world patient care.^31^ Prior to their graduation few dedicated residents and fellows performed the entire HoLEP procedure without an attending physician scrubbing into the case. Such an approach has proved to be safe in many specialties including core‐general surgical procedures and spine surgeries, although it might result in increased operative time.^32^, ^33^ We believe that prospective studies are necessary to further explore the impact of resident involvement on postoperative outcomes, with a focus on the specific HoLEP steps in which they participated.

## 5 CONCLUSION

The HoLEP “En Bloc” technique, with the active participation of residents and fellows, proved to be a feasible, effective and safe procedure for prostates > 200 cc, providing outcomes like those across the literature. However, patients should be counselled on the possible risk of increased operative time and blood transfusion rate.

## AUTHOR CONTRIBUTIONS

**Feres C. Maluf:** Writing/editing manuscript. **Ansh Bhatia:** Data analysis; writing/editing manuscript. **Archan Khandekar:** Project development. **Diana M. Lopategui:** Project development; writing/editing manuscript. **Joao G. Porto:** Project development; writing/editing manuscript. **Ryan R. Chen:** Data collection. **Jean C. Daher:** Data collection. **Mohamadhusni Zarli:** Data collection. **Robert Marcovich:** Project development. **Hemendra N. Shah:** Project development; writing/editing manuscript.

## CONFLICT OF INTEREST STATEMENT

All authors declare that they have no conflicts of interest.
